# 

**Published:** 1894-08-04

**Authors:** 


### CONTENTS.

- **Doctors in Council** - 367

- **Around the Hospitals** - 368

- **On Modern Progress in Ophthalmic Medicine and Surgery.** By Robert Brudenell Carter, F.R.C.S. - 369

- **The Psychologist in History.** Madness or——What? - 370

- **Medical Progress and Hospital Clinics:**

  - Chronic Catarrh of the Middle Ear (*continued*) - 373

  - Medical Progress.—Diseases of Children (*continued*) - 375

  - Progress in Surgery.—Bone, Joint, and Orthopædic Surgery (*continued*) - 377

  - New Appliances and Things Medical - 378

- **Annotations.**—Curiosities of American Lunacy—An Outrage at the British Medical Association—Playgrounds for Little Players 371

- **Notes and News** - 372

- **The Institutional Workshop:**

  - Hospital Construction.—The Convalescent Home of the Royal Infirmary of Edinburgh, Corstorphine - 379

  - Practical Departments - 380

  - Metropolitan Hospital Sunday Fund - 381

  - Construction Notes - 382

  - Hospital Festivals and Meetings - 382

- **Scraps and Gleanings** - 382

### EXTRA SUPPLEMENT.—THE NURSING MIRROR.

- **News from the Nursing World.**—Our Princess—Princess Beatrice at Southampton—The Little King's Holidays—Misplaced Dignity—Our Christmas Competitions—An Excellent Association—Penrith—Nurses at Gateshead—Light Literature—Tea or Tabs?—Within Limits—Aid for French Fishermen—Nothing to Do—Short Items - clxxxi
- **On General Nursing.**—XXIII. Typhoid Fever (*continued*) clxxxiii
- **The Midwives and Trained Nurses' Club** - clxxxiv
- **Amongst the Children.** Isolated or Caged? - clxxxiv
- **Notes from Germany** - clxxxiv
- **Notes from Victoria** - clxxxv
- **Minor Appointments** - clxxxv
- **Appointments** - clxxxvi
- **Everybody's Opinion** - clxxxvi
- **Novelties for Nurses** - clxxxvii
- **A Holiday in Switzerland—A Case for Assistance** clxxxviii
- **The Muses' Looking Glass.** Round Charing Cross Hospital - clxxxix
- **For Reading to the Sick.** God's Compassion - clxc
- **The Annual Meeting of the Royal British Nurses' Association** - clxc
- **Notes and Queries** - clxc

